# Building a web-based tool to support clinical decisions in the control of *Chlamydia trachomatis *and *Neisseria gonorrhoeae *infections

**DOI:** 10.1186/1753-6561-7-S7-S11

**Published:** 2013-12-20

**Authors:** Kun Zhao, Fasheng Qiu, Guantao Chen

**Affiliations:** 1 Department of Mathematics and Statistics, Georgia State University, Atlanta, GA 30303, USA; 2 Department of Computer Science, Georgia State University, Atlanta, GA 30303, USA

**Keywords:** sexually transmitted diseases, mathematical model, optimization, Model-View-Controller architecture, clinical decision support, public health

## Abstract

*Chlamydia trachomatis *(CT) and *Neisseria gonorrhoeae *(GC) are the agents of two common, sexually transmitted diseases afflicting women in the United States (http://www.cdc.gov). We designed a novel web-based application that offers simple recommendations to help optimize medical outcomes with CT and GC prevention and control programs. This application takes population groups, prevalence rates, parameters for available screening assays and treatment regimens (costs, sensitivity, and specificity), as well as budget limits as inputs. Its output suggests optimal screening and treatment strategies for selected at-risk groups, commensurate with the clinic's budget allocation. Development of this tool illustrates how a clinical informatics application based on rigorous mathematics might have a significant impact on real-world clinical issues.

## Introduction and background

*Chlamydia trachomatis *(CT) and *Neisseria gonorrhoeae *(GC) are the etiological agents of the two most commonly reported sexually transmitted diseases (STDs) among women in the United States. In 2011, 1,412,791 cases of sexually transmitted CT infection were reported to the Centers for Disease Control and Prevention (CDC) [1]. This case count corresponds to a rate of 457.6 cases per 100,000 population, an increase of 8% over 2010. A common co-infection with CT [2], GC infection was reported a total number of 321,849 cases, corresponding to a rate of 104.2 cases per 100,000 population [1].

To control the spread of STDs, there are some screening guidelines available to clinics. For example, the CDC recommends annual CT screening for sexually active adolescents and young women [3]. The U.S. Preventive Services Task Force (USPSTF) recommends screening all sexually active women, including those who are pregnant, for gonorrhea, if they are at increased risk for infection [4]. Recent data suggest that screening rates in young women are low, with most young women not getting screened [5,6]. The CDC estimates that the incidence of CT is more than twice the number actually reported [7], at least partly because of low screening rates and the nature of CT infection, which is often asymptomatic. Perhaps another reason is that, detection is typically relegated to public clinics, which may have insufficient budgets to screen all eligible women.

To improve the efficient use of limited clinical resources, mathematical resource allocation models have been developed to calculate an optimal solution regarding the selections of patient groups, screening assays, and treatment regimens [5-7]. The parameters used in these models typically come from published data [8]. However, they may be tailored to any particular demographic environment. Our goal, thus, has been to provide a rigorous mathematical framework, into which the end-user can insert specific parameters, adjusted to reflect local conditions and constraints.

To achieve the goal, our approach employs three steps. First, we have designed a mathematical model as our theoretical foundation to address both CT and GC infections. Second, we have analyzed and interpreted the computational results of the proposed model. Finally, we have implemented the mathematical model as a web-tool in which the local clinical manager is enabled to particularize strategies to local conditions and resources. Previously [8,9], we addressed the first two steps; here we elaborate the final stage.

## Method

### Mathematical formulation

The proposed model is a nonlinear cubic binary model. We briefly introduce the model here; see our previous publications for details [8,9]. The patient population comprises  groups, with  available screening assays,  available treatment regimens with funding limitation . We define the following three decision variables:

$$x_{i}=\left\{\begin{array}{cc} 1 & \text{if}\;\text{patient}\;\text{group}\;i\;\text{is}\;\text{selected}\\ 0 & \text{Otherwise } \end{array}\right)$$

$$y_{j}=\left\{\begin{array}{cc} 1 & \text{if}\;\text{screening assay}\;j\;\text{is}\;\text{selected}\\ 0 & \text{Otherwise } \end{array}\right)$$

$$z_{k}=\left\{\begin{array}{cc} 1 & \text{if}\;\text{treatment regimen}\;k\;\text{is}\;\text{selected}\\ 0 & \text{Otherwise } \end{array}\right)$$

*The objective function is to maximize the likely rate of cured outcomes given the available screening assays and treatment regimens for given patient groups*.

(1) $$Max\underset{i,j,k}{\sum }Pop_{i}\cdot Cur_{ijk}\cdot x_{i}y_{j}z_{k}:=\overset{m}{\underset{i=1}{\sum }}\overset{r}{\underset{j=1}{\sum }}\overset{s}{\underset{k=1}{\sum }}Pop_{i}\cdot Cur_{ijk}\cdot x_{i}y_{j}z_{k}$$

Subject to funding availability

(2) $$\underset{i,j,k}{\sum }Pop_{i}\cdot Cost_{ijk}\cdot x_{i}y_{j}z_{k}\leq b$$

Where $Pop_{i}$ represents the population of the  th group, $Cur_{ijk}$ and $Cost_{ijk}$ represent the expected rate of cured infection cases and the costs, correspondingly, over the population of the  th group using the  th screening assay and treated with  th regimen. Assuming the same screening assay and the same treatment are applied to all patients, we have:

(3) $$\overset{r}{\underset{j=1}{\sum }}y_{j}=1\;and\;\overset{s}{\underset{k=1}{\sum }}z_{k}=1$$

The solutions for the three decision variables give us an optimal strategy, maximizing the expected number of cured cases. This model is nonlinear, and can be converted into a knapsack problem (which is a NP-hard problem) [8,9]. There is no simple, analytic solution to solve this model [10]. Instead we adopt a reasonably efficient, two-step branch-and-bound algorithm to give an exact solution.

### Implementation overview

Our implementation plan is to provide highly configurable and user-friendly, web-oriented software that allows a clinical manager to specify parameters such as prevalence rate, budget availability, and costs. Accepting these user-specified parameters, the tool aims to compute a detailed optimal strategy commensurate with that budget. Additionally the users can explore several scenarios by adding/deleting patient groups, screening assays or treatment regimens.

The application was developed using Java Enterprise Edition (rendering it portable to Windows, Linux, Unix or Mac OS), and employ Model-View-Controller (MVC) architecture and Object-relational (OR) mapping to reduce the amount of code, and Multi-thread programming to speed up computation. Dynamic-HTML (DHTML) is extensively to allow user configuration of any parameter combination of population, screening assays and treatment regimens easily. The application wide data is stored in MySQL, which saves the information of user and superuser. As for this information, only superusers can change the data structure. The same database also stored the parameters for calculating the optimal strategy. For example, each screening assays and regimens (e.g. the sensitivity, specificity, unit costs and etc) based on the publish data is saved as default reference values. It will be loaded automatically as the any user initially login to the tool. The tool also allows users to over-ride these inputs as their local site may have individual scenario (e.g. higher/lower costs of the assays than the default one).

### Architecture and major modules

**Figure 1:**
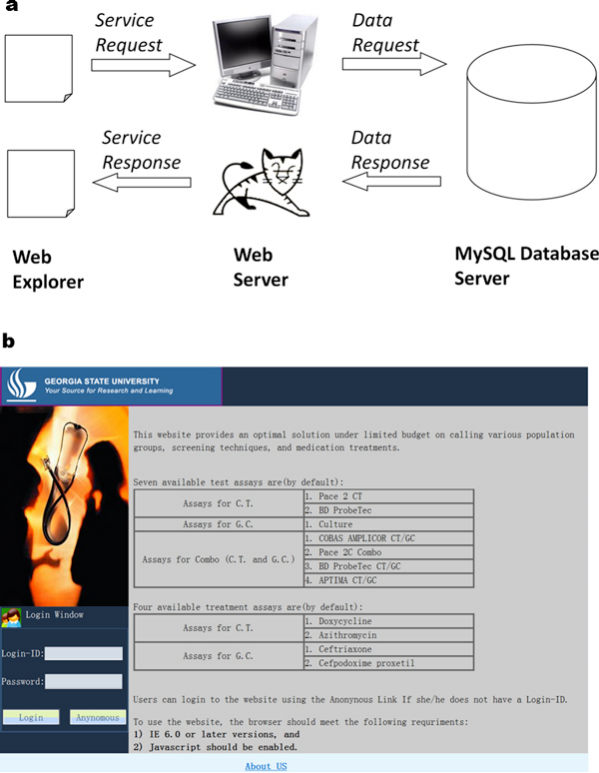
(a) Multi-layer MVC architecture. (b) The welcome page. Two types of users are required.

Our application adopts a multi-layer MVC architecture shown in Figure 1a. From left to right, there are: Web Explorer layer, Web Server layer and MySQL Database layer. The Web Explorer layer includes the web pages (representing View) users use to send service requests and receive service responses. Web Server layer handles all the business logic to process user's requests. This layer also contains a controller component, which accesses application data in MySQL database (not shown in Figure 1). The processed result is stored in model components (JavaBeans) and routed back to the controller component, where it constructs the result page.

The business logic can be categorized into two major modules. The data management module analyzes various application-wide data, such as user information, transaction data, population data, and screening and treatment data. It also identifies the user as anonymous or advanced-users, provides help and retrieval of the default settings. The population, screening and treatment module is used to customize population data by adding/removing a population group. Accordingly, this module then automatically changes the structures of parameter input tables for infection and/or co-infection rates reflected in the population data. It is also used to customize screening and/or treatment choices.

## Results

**Figure 2:**
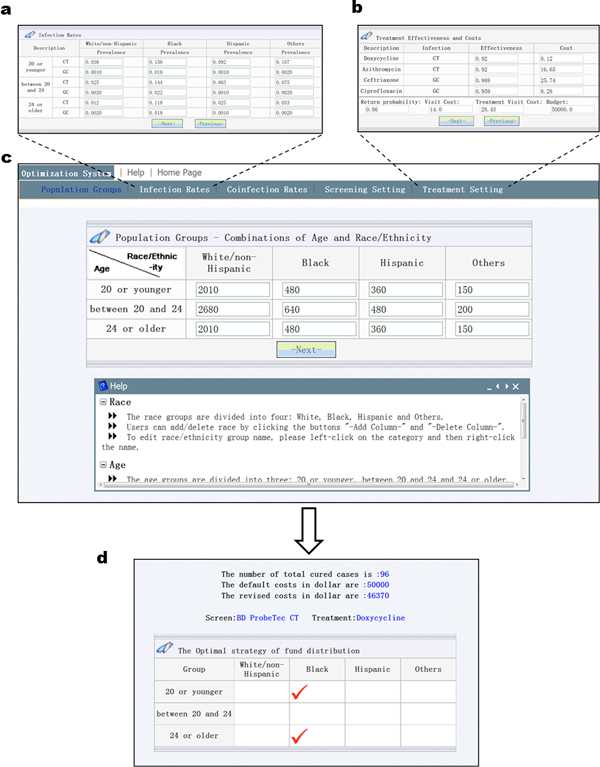
**User interfaces and parameters need to be specified**.

**Figure 3:**
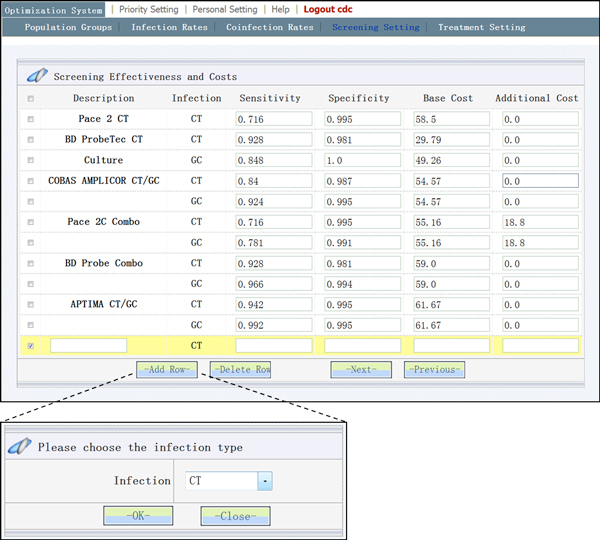
**User may tailor his/her model by adding and deleting the screening assays**.

To achieve the goal of calculating an optimal strategy automatically, the mathematical model is successfully implemented by the new web-based tool (Figure 1b and Figure 2). This web-tool has five main pages: population groups, infection rates, co-infection rates, screening setting and treatment setting. If a clinical manager has difficulties, there are also help windows available to provide tutorial information. These pages follow each other in sequence as a clinical manager submits his/her local parameters. For example, the mathematical variable $x_{i}$ is configured within the "population group" page, where visiting patients are initially divided into 12 groups reflecting different populations at local clinics (Figure 2c). The corresponding local prevalence rate could be specified in the "infection rate" page (Figure 2a). The "co-infection rate" page specifies how likely that the CT patients in the population also have GC. The other two variables $y_{i}$ and $z_{i}$ control the decision on screening assays and treatment regimens, and are specified in the "screening setting" page (Figure 3) and "treatment setting" page (Figure 2b), correspondingly.

After the required parameters for the model are specified, this web-tool calculates the optimal strategy by solving the proposed mathematical model with the accurate, two-step branch-and-bound algorithm. An example of a calculated optimal solution is shown in Figure 2d. It is interpreted as follows: after the local situation at a clinic is specified, the optimal solution recommends screening the black groups 20 or younger and 24 or older using BD ProbeTec CT, and to treat those showing positive screening results with doxycycline. This tool also reports: given a pre-determined budget of $50,000 (default costs in Figure 2d), the plan suggested by the model can be expected to cure 96 patients given the local CT and GC prevalence rates. Furthermore, this tool also suggests that to achieve the expected cures, $46,370 (revised costs in Figure 2d) should be sufficient.

The other part of the goal is to allow clinical experts to re-design the decision model. Several steps are needed to achieve this. First, a login page was designed to classify users as "anonymous" or "advanced". Anonymous users are not required to have passwords to use this tool, and they can access the basic functions of the tool needed to calculate the optimal solutions as we describe above (Figure 2). To re-design the decision model, clinical professionals have to be authenticated as advanced-users. An advanced-user can add or delete population groups, screening assays and treatment regimens. The total number of underlying decision variables $x_{i}$, $y_{i}$ and $z_{i}$ are updated correspondingly.

For example, Figure 3 illustrates a feature available to advanced-users, namely the addition of a new screening assay, including whether the assay is for CT or GC or both (this is accomplished in a pop-up window). After the advanced-user has added a new screening assay to the model, the tool will re-calculate the model taking the addition diagnostic assay into consideration, by augmenting the terms of decision variable $y_{i}$. Analogously, adding/deleting population groups and treatment regimens will lead to a re-calculation with respect to changes in parameters related to $x_{i}$ and $z_{i}$(the interface webpage is not shown). The "re-design" features give advanced-users flexibility to re-model new situations and to tailor the computation efficiently to his or her specific situation.

## Discussion

Many efforts towards improvements of the quality of health care have resulted in the development of clinical decision-supports systems [11-13]. However, clinical practitioners seem prone to rely on their own experience to solve problems instead of using decision aids [11,14]. The barrier is due in part to the fact that practice guidelines (typically promulgated by organizations like the CDC) do not fit local clinical situation; this is certainly true in the case of sexually transmitted disease control programs.

Significantly different from other clinical decision support systems [11,15,16], this new web-based tool is designed to lower that barrier by enabling practical-minded, clinical managers to impose their view of local realities and still avail themselves of a rigorous mathematical model for the number-crunching. This is accomplished without compromising ease of use, thanks to its user friendly interfaces and didactic instructions for adding or deleting new population groups, screening assays or treatment regimens. This design not only allows users to do "what-if" analysis, by manipulating the mathematical model with their own parameters, but also gives flexibility to accomplished users to re-parameterize the model virtually from scratch. To our knowledge, this is the first web-based tool (which utilizes a rigorous mathematical model) to offer a detailed, optimal strategy to select at-risk patient groups, as well as screening assays and treatment regimens for the control and prevention of CT and GC - all within a specified budgetary constraint.

Of course, there are limitations to the approach and challenges in its implementation. First, the new decision tool depends on the underlying mathematical model, which embodies necessary assumptions. Though parameters can be adjusted, the underlying assumptions are fixed. Second, there is a theoretical computational limit while solving the model. For example, the complexity of the two-step branch-and-bound algorithm to the model has an overall running time of *O(n·m2^m^)*, where *n *is the number of the combinations of screening and treatment strategies satisfying conditions (3), and *m *is the number of population groups [8]. As the number of division in population groups, the choices of screening assays, and the availability of treatment regimens increase, the computational challenge increases. We are optimistic about overcoming the computational challenge for following reasons. The values of *m *and *n *are not huge numbers in reality. The availability of regimens determines the value of *n*. There are usually practical guidelines at each clinic, regarding how to partite patients into *m *groups. Commercial software applications may adopt approximation algorithms for solving the proposed model, too. For example, Excel Solver's approximation algorithm sometimes calculates near-optimal solutions, while the two-step branch-and-bound algorithm is an exact algorithm which always calculates the optimal solution. We demonstrated the advantage of using the two-step branch-and-bound algorithm over Excel Solver's approximation algorithm, in term of the computational accuracy and the running time [8]. A third challenge is to provide a reasonably quick service response -- an important factor for users expecting a timely browsing experience. To overcome this obstacle and we have designed a dedicated logic handler on the basis of a multi-thread programming technique. We provide detail on threading techniques in the Appendix. The computation time of the algorithm thus becomes a matter of seconds [8]. Cutting edge technology and advanced algorithm design rise to meet the computational challenge and to satisfy user expectation of a quick response. Fourth, we are aware that the new application needs to be tested and evaluated by clinical managers so that it can be improved, both with respect to its user-interfaces and its back-end algorithm. We are currently actively seeking collaboration with clinicians to evaluate this tool. A short follow-up report of actual use will be ready once we have a beta testing within a clinic and across sites evaluations could be also reported after we receive feedback from more clinics.

Hopefully, with cooperative interaction between clinician and mathematician, these limitations can be ameliorated, resulting in an improved tool. We are optimistic that successful implementation of this tool will highlight the feasibility of applying complicated mathematical models to practical clinical problems via a powerful informatics approach.

## Competing interests

The authors declare that they have no competing interests.

## Appendix

The algorithm of the logical handler is sketched in Table 1 (for master thread) and Table 2 (for slave threads).

**Table 1:** The algorithm for the master thread

**Procedure **master (*groups*, *screenings*, *treatments*) (*screeningsCT*, *screeningsGC*) = identify the list of screening plans for CT and GC, respectively. (*treatmentsCT*, *treatmentsGC*) = identify the list of treatment plans for CT and GC, respectively. FOR I = 1 TO *screeningsCT*_size FOR J = 1 TO *screeningsGC*_size FOR K = 1 TO *treatmentsCT*_size FOR L = 1 TO *treatmentsGC*_size Create a combination of *screeningsCT[I], screeningsCT[J], treatmentsCT[K]*, and *treatmentsGC[L]*. Categorize all combinations into different types. Distribute each type of combinations into a slave thread. Wait for slave threads to finish the computation. Collect all results and calculate the final optimal result. Return the optimal result to logic handler. **End procedure **master.

**Table 2:** The algorithm for slave threads

**Procedure **slave(*groups*, *screenings*, *treatments, combinations, budget*) FOR I = 1 TO *combinations*_size Get screening1, screening2 from *combinations*[I] and *screenings*; Get treatment1, treatment2 from *combinations*[I] and *treatments*; FOR J = 1 TO *groups*_size Update *combinations*[I] by adding the number of cured people in *groups*[J] given screening1, screening2, treatment1, and treatment2. Update *combinations*[I] by adding the cost for curing people in *groups*[J] given screening1, screening2, treatment1, and treatment2. Run knapsack algorithm to get the local optimal results for this type of combinations under *budget*. Return the local optimal results to the master thread. **End procedure **slave.

Note that in Table 1, after all combinations are created, they are categorized into different types. For example, one of the types is ct-single-screening-single-treatment, which stands for the combination of screening and treatment plan for CT where both the screening and treatment plan are a single plan (only used for screening/treating a single disease). After all types are created, they are distributed into slave threads (one type is processed by each a slave thread) to calculate the number of cured people among the population groups. The processing result is fed back into the master thread. The algorithm for slave threads is described in Table 2.

After calculating the number of people expected to be cured, as well the associated cost of the given type of combinations, the optimal results are obtained by solving several "knapsack" problems. For insight on how to convert this mathematical model into knapsack problems and the details of two-step branch-and-bound algorithm, please refer our previous publication. [8]

## Declarations

This project is supported in part by the GSU MBD program and a GSU mathematics assistantship.

This article has been published as part of *BMC Proceedings *Volume 7 Supplement 7, 2013: Proceedings of the Great Lakes Bioinformatics Conference 2013. The full contents of the supplement are available online at http://www.biomedcentral.com/bmcproc/supplements/7/S7.
